# Comparative Profiling of Primary Colorectal Carcinomas and Liver Metastases Identifies LEF1 as a Prognostic Biomarker

**DOI:** 10.1371/journal.pone.0016636

**Published:** 2011-02-24

**Authors:** Albert Y. Lin, Mei-Sze Chua, Yoon-La Choi, William Yeh, Young H. Kim, Raymond Azzi, Gregg A. Adams, Kristin Sainani, Matt van de Rijn, Samuel K. So, Jonathan R. Pollack

**Affiliations:** 1 Department of Medicine, Santa Clara Valley Medical Center, San Jose, California, United States of America; 2 Department of Medicine, Stanford University, Stanford, California, United States of America; 3 Department of Surgery and Asian Liver Center, Stanford University, Stanford, California, United States of America; 4 Department of Pathology, Stanford University, Stanford, California, United States of America; 5 Department of Pathology, Samsung Medical Center, Sungkyunkwan University School of Medicine, Seoul, Korea; 6 Department of Pathology, Santa Clara Valley Medical Center, San Jose, California, United States of America; 7 Department of Surgery, Santa Clara Valley Medical Center, San Jose, California, United States of America; 8 Division of Epidemiology, Health Research and Policy, Stanford University, Stanford, California, United States of America; National University of Singapore, Singapore

## Abstract

**Purpose:**

We sought to identify genes of clinical significance to predict survival and the risk for colorectal liver metastasis (CLM), the most common site of metastasis from colorectal cancer (CRC).

**Patients and Methods:**

We profiled gene expression in 31 specimens from primary CRC and 32 unmatched specimens of CLM, and performed Significance Analysis of Microarrays (SAM) to identify genes differentially expressed between these two groups. To characterize the clinical relevance of two highly-ranked differentially-expressed genes, we analyzed the expression of secreted phosphoprotein 1 (SPP1 or osteopontin) and lymphoid enhancer factor-1 (LEF1) by immunohistochemistry using a tissue microarray (TMA) representing an independent set of 154 patients with primary CRC.

**Results:**

Supervised analysis using SAM identified 963 genes with significantly higher expression in CLM compared to primary CRC, with a false discovery rate of <0.5%. TMA analysis showed SPP1 and LEF1 protein overexpression in 60% and 44% of CRC cases, respectively. Subsequent occurrence of CLM was significantly correlated with the overexpression of LEF1 (chi-square *p* = 0.042), but not SPP1 (*p* = 0.14). Kaplan Meier analysis revealed significantly worse survival in patients with overexpression of LEF1 (*p*<0.01), but not SPP1 (*p* = 0.11). Both univariate and multivariate analyses identified stage (*p*<0.0001) and LEF1 overexpression (*p*<0.05) as important prognostic markers, but not tumor grade or SPP1.

**Conclusion:**

Among genes differentially expressed between CLM and primary CRC, we demonstrate overexpression of LEF1 in primary CRC to be a prognostic factor for poor survival and increased risk for liver metastasis.

## Introduction

Despite considerable progress in the diagnosis and treatment of colorectal cancer (CRC) over the last few decades leading to a significant decline in cancer-related mortality,[1], [2] CRC remains a major public health problem throughout the world. In the United States, CRC is the third most common cancer and is also the third leading cause of cancer death in men and women combined.[3]

Worldwide it represents the third most common cancer and second most common cause of cancer-related death.[4] Once metastasis has occurred in CRC, a complete cure of the disease is unlikely. Therefore, there is a need for better understanding of the molecular mechanisms underlying the metastatic phenotype that may provide information leading to the development of drugs to control or prevent metastatic disease.[1]

Colorectal liver metastasis (CLM), occurring in about 60% of CRC patients during the course of their treatment, is the most common distant metastasis from CRC. Several clinical prognostic factors, such as lymph node status and size of the primary tumor, have been identified for CLM.[5], [6] However, little is known about the prognostic significance of molecular markers for CLM.

Recent development and application of human genome and high-throughput technologies, such as DNA microarrays, allows us to simultaneously examine thousands of genes, leading to a much better understanding of carcinogenesis - a great step toward individualized personal medicine.[7] Published studies on CRC gene expression profiling have mainly examined normal vs. tumor tissues or different stages of CRC,[8], [9] or treatment outcomes by the differences in gene expression profiling.[10], [11] To identify molecular markers of clinical significance, we used DNA microarrays to compare the gene-expression profiles of specimens from primary CRC and specimens from CLM. We reasoned that genes upregulated in metastasis might also be relatively overexpressed in a subset of clinically-aggressive primary CRC. After identifying upregulated CLM-signature genes, we used tissue microarrays (TMAs) to study the protein expression of selected signature genes in an independent cohort of primary CRC, to correlate their expression with clinical significance and outcome.

## Results

### Identification of gene signatures distinguishing CLM from primary CRC by expression profiling

**Figure 1:**
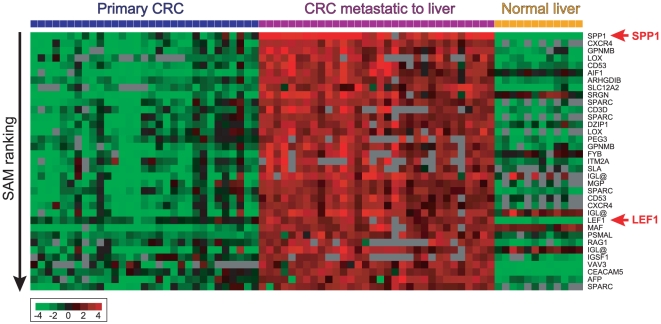
Heatmap showing the top 35 ranking genes, based on SAM analysis, with greatest increased expression in CRC with CLM compared to primary CRC or normal liver. Rows represent individual genes and columns represent individual tissue samples. In each tissue sample, the log_2_ ratio of abundance of transcripts of each gene relative to its mean abundance across all tissue samples is depicted according to the color score shown at the bottom. Grey indicates missing or excluded data.

To survey the differentially expressed genes between CLM and primary CRC (also compared to normal liver, a potential tissue contaminant of CLM), we used cDNA microarrays containing ∼19,500 unique genes to profile the gene expression in 31 primary CRC specimens from 30 patients, and 32 unmatched CLM specimens from 31 patients who underwent liver resection. We then performed supervised analysis using SAM (with a Student's t-test metric) and identified 1,186 discriminatory cDNAs (corresponding to 963 unique genes) with significantly higher expression in CLM when compared to primary CRC, and to normal liver (previously profiled,[12]), with a false discovery rate (FDR) of <0.005% (Table S2). The top 35 differentially expressed genes are shown in Fig. 1. The 20 highest-ranking genes were *SPP1, CXCR4, GPNMB, LOX, CD53, AIF1, ARHGDIB, SLC12A2, PRG1, SPARC, CD3D, DZIP1, PEG3, FYB, ITM2A, SLA, IGLC2, MGP, LEF1,* and *MAF*. Similar results were obtained using SAM with a non-parametric, Wilcoxin rank-based analysis (Table S3).

### LEF1 protein overexpression in CRC correlates with CLM and overall survival

**Figure 2:**
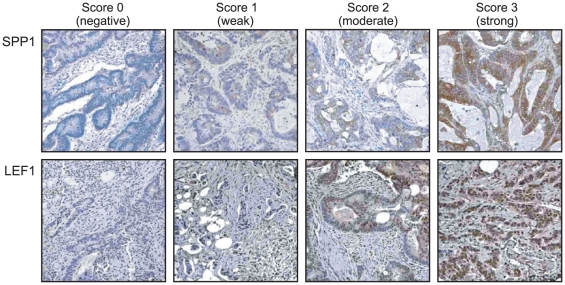
Immunohistochemical staining and assessment of SPP1 or LEF1 in representative core sections from the CRC tissue microarray. Staining of SPP1 and LEF1 were respectively visualized in the cytoplasm and nuclei of cancer cells (X 400 magnification).

Primary CRC specimens with relative increased expression of CLM signature genes might exhibit increased metastatic potential. To further study the significance of such potential biomarkers identified *via* SAM analysis, we examined the protein expression of two highly ranked and biologically plausible signature genes (for which IHC-validated antibodies were also available), SPP1 (linked to metastasis[13]) and LEF1 (lymphoid enhancer factor-1; involved in WNT signaling[14]), in an independent TMA cohort of CRC specimens. Examples of antibody staining by intensity are shown in Fig. 2. Correlation of SPP1 or LEF1 overexpression (IHC scored +2 or +3) with subsequently occurring CLM is shown in Table 1. LEF1 overexpression was significantly correlated with CLM (Fisher's exact test, *p* = 0.042). No significant correlation was observed between SPP1 overexpression and subsequent CLM.

**Table 1:** Correlation between liver metastasis and SPP1 or LEF1 overexpression^a^.

	SPP1 Overexpression (No. of patients)			LEF1 Overexpression (No. of patients)		
Liver metastasis	Yes	No	*p*	Yes	No	*p*
Yes	24	10		16	10	
No	66	51		41	63	
			0.14			0.042

a As a predictor for liver metastasis, the sensitivity, specificity, positive predictive value, and negative predictive value for overexpression of SPP1 in the primary colon cancer tissue were 26.7%, 83.6%, 70.6%, and 70.6%, respectively. In contrast, the sensitivity, specificity, positive predictive value, and negative predictive value for overexpression of LEF1 are 28.1%, 86.3%, 61.5%, and 60.6%, respectively. Despite that the sensitivity and specificity were higher in LEF1, both showed modest performance.

**Figure 3:**
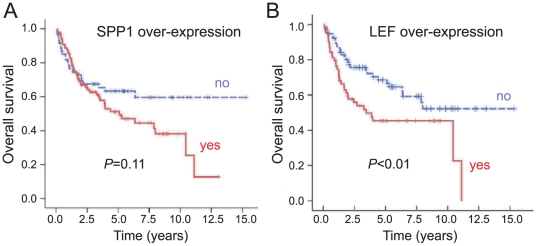
Kaplan-Meier overall survival curves based on (A) SPP1 overexpression (log rank *p* = 0.11) or (B) LEF1 overexpression (log rank *p*<0.01).

We used Kaplan-Meier analyses to investigate the impact of SPP1 or LEF1 overexpression on overall survival. LEF1 overexpression was significantly associated with worse survival (log-rank *p*<0.01; Fig. 3B). In contrast, SPP1 overexpression was not significantly correlated with a worse outcome, though there was a trend toward poor survival (log-rank *p* = 0.11; Fig. 3A)

### LEF1 is a significant prognostic factor in multivariate analysis

As expected, higher stage was found to be significantly associated with worse overall survival. In the univariate model, the hazard ratios (HRs) for stage 2, 3, and 4 vs. 1 was 1.63, 3.47 (*p* = 0.02) and 12.63 (*p*<0.001), respectively; in the multivariate model, the HRs are 1.74, 3.74 (*p* = 0.01), and 13.74 (*p*<0.001), respectively. The HRs were also statistically significant for LEF1 overexpression in both the univariate and multivariate models - 1.78 (*p*<0.05) and 1.66 (*p*<0.05), respectively. Neither SPP1 overexpression nor tumor grade was significantly associated with survival in either analysis. Table 2 summarizes the HRs and 95% confidence intervals for the variables in both models.

**Table 2:** Univariate and Multivariate Hazard Ratios From Cox Proportional Hazards Regression Models.

Variable	*P*	Hazard Ratio	95% Confidence interval
Univariate analysis			
LEF1	0.03	1.66	1.04–2.63
SPP1	0.37	1.25	0.77–2.02
Stage			
1*			
2	0.36	1.63	0.57–4.63
3	0.02	3.47	1.27–9.50
4	<.0001	12.63	4.86–32.84
Tumor grade			
1*			
2	0.60	0.69	0.17–2.83
3	0.96	1.04	0.24–4.52
Multivariate analysis			
LEF1	0.02	1.78	1.09–2.89
SPP1	0.54	0.85	0.51–1.43
Stage			
1*			
2	0.31	1.74	0.60–5.02
3	0.01	3.74	1.35–10.39
4	<.0001	13.74	5.19–36.36
Tumor grade			
1*			
2	0.48	0.59	0.14–2.54
3	0.82	0.84	0.19–3.76

*Reference category.

## Discussion

In our study, we sought to identify signatures of metastasis embedded in a subset of primary tumors, which might predict clinically-aggressive behavior [15]. Using supervised SAM analysis, we identified 963 unique genes that are significantly overexpressed in CLM vs. primary CRC (and potentially contaminating normal liver tissue). In an independent set of tissue microarrays, we examined two highly-ranked genes (LEF1 and SPP1) as surrogate biomarkers for the CLM signature, and demonstrated that overexpression of LEF1, but not SPP1, in the primary CRC tissues correlates with a statistically significant increased risk of CLM, albeit its sensitivity and specificity in predicting liver metastasis were modest. In addition, independent of tumor stage, overexpression of LEF1, not SPP1, denotes a poor prognosis for survival.

LEF1 was initially identified as a pre-B and T-lymphoid-specific gene encoding a DNA-binding protein of high mobility group (HMG) proteins.[16], [17] It is a member of the T-cell factor/lymphoid-enhancing factor (TCF/LEF) family of transcription factors, which acts through the Wnt signaling pathway[18], [19], [20] to regulate gene expression and coordinate many cellular processes in normal development and tissue homeostasis, and, when deregulated, in colonic tumorogenesis and metastasis. Upon Wnt stimulation, LEF1 or other TCF/LEF-family transcription factors associate with β-catenin, a key cytoplasmic/nuclear mediator of Wnt pathway, and activate Wnt-responsive target genes. In contrast, without Wnt stimulation, glycogen synthase kinase (GSK)-3 (in a complex with APC) constitutively phosphorylates β-catenin, resulting in its proteasome-dependent degradation.[21] Although genetic and epigenetic changes have been documented in several targets throughout the pathway, mutation in either APC or β-catenin appears to be a crucial element in CRC carcinogenesis.[22] The LEF1 gene itself is not normally expressed in the adult intestinal epithelium, but only observed in the embryos while development is in progress. However, its overexpression has been well documented in CRC tumorigenesis[23], and denotes aberrant activation of the Wnt/β-catenin pathway *via* stabilization of LEF1/β-catenin complex. Indeed, LEF1 is a direct transcriptional target of the LEF1/β-catenin complex[23], indicating a positive feedback loop for Wnt signaling, and suggesting LEF1 might be useful as a prognostic biomarker of Wnt pathway activation. Our findings are consistent with a prior study showing nuclear (active) β-catenin staining to be prognostic in colon cancer,[24] and highlight a role of Wnt signaling in colon cancer progression and liver metastasis.

As the prefix "osteo" suggests, osteopontin (or OPN, also known as secreted phosphoprotein 1 [SPP1], bone sialoprotein I, early T-lymphocyte activation 1) was initially recognized as an important glycosylated, adhesive phosphoprotein in bone.[25] Since then, several lines of evidence have shown its role in controlling tumorigenicity, progression and metastasis *via* its diverse ability as a cell-matrix mediator to interact with a variety of factors such as cell surface receptors (integrins and CD44), secreted proteases (matrix metalloproteinases and urokinase plasminogen activator), and growth factor/receptor pathways (TGF/EGFR and HGF/Met).[26] Overexpression of SPP1 has been reported in several human cancers, including lung, breast and colon cancers. In a gene-expression profiling study, Agrawal *et al* identified SPP1 as a lead marker correlating with CRC progression, and strongly expressed in CLM.[27], [28], [29] Most recently, Rohde *et al* observed that overexpression of SPP1 is indicative of poor survival in CRC and is significantly correlated with CLM.[30] In addition, overexpression of SPP1 correlates with increased immunohistochemical staining of β-catenin and, in an *in vivo* model, with Wnt activating mutations. These data suggest a crucial role of SPP1 in CRC progression and metastasis likely *via* molecular cross-talk with the Wnt pathway. Our results partially corroborate with published data in that we observe a trend towards poorer survival and CLM with SPP1 overexpression, though it is not statistically significant. This difference may be due to a smaller sample size and/or shorter follow-up time. Nonetheless, our data suggest that the overexpression of LEF1 is a stronger prognostic factor than SPP1 in correlating overall survival and CLM.

Though our analysis focused primarily on LEF1 and SPP1, other highly-ranked signature genes with increased expression in CLM compared to primary CRC also have biological functions consistent with roles in tumor progression, and might have prognostic utility. For example, CXCR4 (Chemokine (C-X-C motif) receptor 4) has been implicated in breast [31] and colon cancer metastasis.[32] LOX (lysyl oxidase) was shown to be associated with hypoxia where it functions in metastasis and predicts poor outcome in breast cancer.[33] SPARC (secreted protein, acidic, cysteine-rich; osteonectin) was identified among genes that mark and mediate breast cancer metastasis.[34] Further studies are needed to characterize these and other signature genes in CRC progression.

Much of the published data comparing gene expression profiles from primary CRC vs. CLM have reported the differences in up- or down-regulated genes.[35], [36], [37], [38], [39], [40], [41], [42], [43], [44] To the best of our knowledge, our data are the first to identify the expression of LEF1 as a predictor of overall survival, and an indicator of CLM. Comparisons of primary CRC vs. CLM, or of primary CRC associated with or without CLM, have identified gene signatures with relevance to colorectal cancer progression.[27], [30], [35], [36], [37], [38], [39], [40], [41], [43] However, there are minimal overlaps between our CLM signature genes and the top-ranking CLM/progression-associated genes reported in these studies (Table 3). Several issues may explain the discordance among studies, such as: variability in patient cohorts, technical differences (the composition of the microarrays used, study design, statistical methodologies), and variability in the use of independent cohorts of patients to validate candidate prognostic genes.[45]

**Table 3:** Overlap of CLM genes with top-ranking CLM/progression genes from other published studies.

Gene Symbol	Overlapping with Other Studies	Gene Name
AGR2	Ki et al.[35], Tackels-Horne et a.[40]	ANTERIOR GRADIENT 2 HOMOLOG (XENOPUS LAEVIS)
CD44*	Ki et al.[35], Lin et al. [38]; Takayama et al.[47],	CD44 ANTIGEN (INDIAN BLOOD GROUP)
CDC2	Ki et al.[35], Takahashi et al.[45]	CELL DIVISION CYCLE 2, G1 TO S AND G2 TO M
CDH17	Ki et al.[35], Tackels-Horne et a.[40]	CADHERIN 17, LI CADHERIN (LIVER-INTESTINE)
CEACAM5	Ki et al.[35], Tackels-Horne et a.[40]	CARCINOEMBRYONIC ANTIGEN-RELATED CELL ADHESION MOLECULE 5
CEACAM6	Ki et al.[35], Tackels-Horne et a.[40]	CARCINOEMBRYONIC ANTIGEN-RELATED CELL ADHESION MOLECULE 6 (NON-SPECIFIC CROSS REACTING ANTIGEN)
CKS2	Li et al.[37], Lin et al. [38]	CDC28 PROTEIN KINASE REGULATORY SUBUNIT 2
EFEMP1	Ki et al.[35]	EGF-CONTAINING FIBULIN-LIKE EXTRACELLULAR MATRIX PROTEIN 1
HNRPA1	Ki et al.[35], Li et al.[37]	HETEROGENEOUS NUCLEAR RIBONUCLEOPROTEIN A1
MAD2L1	Ki et al.[35], Li et al.[37]	MAD2 MITOTIC ARREST DEFICIENT-LIKE 1 (YEAST)
MMP2	Ki et al.[35], Takayama et al.[43];	MATRIX METALLOPEPTIDASE 2 (GELATINASE A, 72KDA GELATINASE, 72KDA TYPE IV COLLAGENASE)
S100P	Ki et al.[35], Li et al.[37]	S100 CALCIUM BINDING PROTEIN P
SPP1	Agrawal et al.[27], Rohde et al.[30 87]	SECRETED PHOSPHOPROTEIN 1 (OSTEOPONTIN, BONE SIALOPROTEIN I, EARLY T-LYMPHOCYTE ACTIVATION 1)
TIMP1*	Ki et al.[35], Takayama et al.[43]; Takahashi et al.[41]	TIMP METALLOPEPTIDASE INHIBITOR 1
TOP2A	Ki et al.[35], Takahashi et al.[41]	TOPOISOMERASE (DNA) II ALPHA 170KDA
VAV3	Ki et al.[35], Kleivi et al.[36]	VAV 3 ONCOGENE

Gene names are according to the DAVID Bioinformatics Database.

http://david.abcc.ncifcrf.gov/tools.jsp.

*indicates genes present in three studies.

In conclusion, our study shows that overexpression of LEF1 in primary CRC correlates with a higher risk of CLM and denotes poor overall survival. It is a stronger predictor than SPP1, a marker reported in previous transcriptome studies. High-throughput gene expression profiling technology has revealed new insights into the molecular heterogeneity of CRC and identified new and better molecular markers for risk stratification. This holds promise for personalized medicine and improved targeted therapy. To achieve these goals, further studies are needed to understand the functional roles and clinical implications of LEF1, SPP1 and other signature genes for CLM.

## Materials and Methods

### Patients and Tumor Specimens

Freshly frozen CRC specimens (from Santa Clara Valley Medical Center) and CLM specimens (from Stanford University Medical Center) were used for gene expression profiling analysis. We used 31 primary CRC specimens from 30 patients (14 males and 16 females; age range: 36 to 80; stage I/II/III/IV = 1/10/11/8, diagnosed between 2000 and 2004), and 32 CLM specimens from 31 patients (16 males and 15 females; age range: 40 to 82). Tissue microarrays were constructed with paraffin blocks from 154 CRC cases (84 males and 70 females; age range: 27 to 92; stage I/II/III/IV = 26/46/38/44) with median follow-up of 3.0 years (range: 0.6–15.3 years). Study protocols were approved by Institutional Review Board (IRB) both at Stanford University Medical Center (IRB # 12473) and Santa Clara Valley Medical Center, a Stanford-affiliated teaching hospital, (IRB #07/28/00-03 and #2/22/2002-04). Individual informed consent was obtained from all participants involved in the gene expression profiling protocols and was waived in the TMA study by IRB due to the use of coded data and retrospective nature of the study. Clinical characteristics of the patient cohorts are summarized in Table S1.

### Gene expression profiling

To confirm that the sample was representative of the case, a frozen section from each specimen was first prepared and examined. Tissue was then homogenized in Trizol reagent (Invitrogen, Carlsbad, CA), and total RNA isolated per the manufacturer's protocol. RNA quality was assessed by gel electrophoresis. Gene expression profiling was performed as described previously.[46] Briefly, using microarrays of complementary DNA (cDNA) (manufactured by the Stanford Functional Genomics Facility) containing ∼40,000 nonredundant cDNA clones, representing ∼19,500 unique UniGene clusters (i.e., genes), we hybridized Cy5-labeled total RNA from the tumor specimens, along with Cy3-labeled universal reference mRNA (pooled from 11 different cancer cell lines). We imaged arrays using an Axon GenePix 4000B scanner (Molecular Devices, Sunnyvale, CA), extracted fluorescence ratios (ratio of the specimen value to the reference value) using the GenePix software, and entered the data into the Stanford Microarray Database[47] for subsequent analysis. The microarray data are accessible from the Gene Expression Omnibus (Accession GSE22834). cDNA microarray expression data from normal liver specimens were previously published.[12]

### TMA construction and immunohistochemistry

A tissue arrayer (Beecher Instruments, Sun Prarie, WI) was used to construct a primary CRC tissue microarray as described,[48] comprising of 154 primary colorectal tumors each represented by two 6-mm cores. A 4-µm section was cut from the tissue microarray block, deparaffinized in Citrisolv (Fisher Scientific, Hampton, NH) and hydrated in a graded series of alcohol solutions.

For immunohistochemical staining (IHC), anti-osteopontin (SSP1) mouse monoclonal antibody (Novocatra, Newcastle, UK) and anti-LEF1 (lymphoid enhancer factor-1) rabbit polyclonal antibody (Abcam, Cambridge, MA) were used at 1∶100 and 1∶500 dilutions respectively, and incubated overnight at 4°C. Chromogenic detection was then done using a peroxidase-conjugated secondary antibody and DAB reagents provided with the Envision detection kit (DAKO, Carpinteria, CA). SPP1 expression was scored as positive if distinct cytoplasmic staining was present in more than 10% of tumor cells. LEF1 expression was scored as positive when distinct nuclear staining was present in more than 10% of tumor cells. Weak cytoplasmic staining of LEF1 was not counted. The staining intensity was graded on a semiquantitative score (0, negative; 1+, weak; 2+, moderate; and 3+, strong). Survival analysis was performed in two groups depending on the score: overexpression (2+ and 3+) vs. the remainder (0 or 1+). Immunostains were scored by two pathologists (M.vdR. and YLC) blinded to the clinical data.

### Statistical Analysis

For cDNA microarray data, ratios were globally normalized by array and median-centered by gene. We included for analysis the 4,824 cDNAs (corresponding to 3,413 unique genes) that were well-measured (intensity/background >2 in either the test or reference channel) in at least 50% of samples, and variably expressed (>4-fold change from the median) in at least 3 samples. Two-class Significance Analysis of Microarrays (SAM) [49] was used to identify genes that were differentially expressed in CLM compared to primary CRC and normal liver (a potential contaminant of CLM), with statistical significance assessed by a false discovery rate (FDR).

For clinicopathological data, a chi-square test or Fisher's exact test (two-tailed) was used to compare differences in categorical variables across patient groups. Kaplan-Meier methods were used to estimate overall survival. We used both univariate and multivariate Cox proportional hazard regression model to assess the prognostic independence of variables (LEF1, SPP1, stage and tumor grade) for survival. Statistical analyses were performed with the SAS System software, release 9.1.3 (SAS Institute Inc., Cary, NC).

## Supporting Information

Table S1 Summary of clinical characteristics of CRC cohorts.
(XLS)

Table S2 Ranked list of significant (FDR<0.005%) SAM genes (Student's t-test) upregulated in CLM vs. primary CRC and normal liver.
(XLS)

Table S3 Ranked list of significant (FDR<0.005%) SAM genes (Wilcoxin rank) upregulated in CLM vs. primary CRC and normal liver.
(XLS)
